# Distinctive tics suppression network in Gilles de la Tourette syndrome distinguished from suppression of natural urges using multimodal imaging

**DOI:** 10.1016/j.nicl.2018.09.014

**Published:** 2018-09-19

**Authors:** Sandra M.A. van der Salm, Johan N. van der Meer, Daniëlle C. Cath, Paul F.C. Groot, Ysbrand D. van der Werf, Eelke Brouwers, Stella J. de Wit, Joris C. Coppens, Aart J. Nederveen, Anne-Fleur van Rootselaar, Marina A.J. Tijssen

**Affiliations:** a Department of Neurology and Clinical Neurophysiology, Amsterdam UMC, University of Amsterdam, Amsterdam, the Netherlands; b Brain Center Rudolf Magnus, Department of Neurology and Neurosurgery, University Medical Center Utrecht, the Netherlands; c Department of Clinical & Health Psychology, University of Utrecht, GGz Drenthe, Department of Psychiatry, University Medical Center Groningen, the Netherlands; d Department of Radiology, Amsterdam UMC, University of Amsterdam, Amsterdam, the Netherlands; e Department of Anatomy and Neurosciences, Amsterdam UMC, Vrije Universiteit Amsterdam, Amsterdam, the Netherlands; f Netherlands Institute for Neuroscience, An Institute of the Royal Netherlands Academy of Arts and Sciences, Amsterdam, the Netherlands; g Department of Pediatrics/Child Neurology, Neuroscience Campus Amsterdam, Amsterdam UMC, Vrije Universiteit Amsterdam, Amsterdam, the Netherlands; h Department of Psychiatry, Amsterdam UMC, Vrije Universiteit Amsterdam and GGZ inGeest, Amsterdam, the Netherlands; i BIC: Brain Imaging Center, Amsterdam UMC, University of Amsterdam, Amsterdam, the Netherlands; j Department of Neurology, University Medical Centre Groningen, University of Groningen, the Netherlands; k Stichting Epilepsie Instellingen Nederland (SEIN), Zwolle, the Netherlands

**Keywords:** Gilles de la Tourette syndrome, Functional magnetic resonance imaging, Suppression, Tics, Urges

## Abstract

**Background and objectives:**

Gilles de la Tourette syndrome (GTS) is a neuropsychiatric disorder characterized by tics. A hallmark of GTS is the ability to voluntarily suppress tics. Our aim was to distinguish the neural circuits involved in the voluntary suppression of ocular tics in GTS patients from blink suppression in healthy subjects.

**Methods:**

Fifteen GTS patients and 22 healthy control subjects were included in a multimodal study using eye-tracker recordings during functional MRI (fMRI). The ability to suppress tics/blinks was compared both on subjective (self-rating) and objective (eye-tracker) performance. For fMRI analysis we used a novel designed performance-adapted block design analysis of tic/blink suppression and release based on eye-tracker monitoring.

**Results:**

We found that the subjective self-reported ability to suppress tics or blinks showed no significant correlation with objective task performance. In GTS during successful suppression of tics, the dorsal anterior cingulate cortex and associated limbic areas showed increased activation. During successful suppression of eye blinks in healthy subjects, the right ventrolateral prefrontal cortex and supplementary and cingulate motor areas showed increased activation.

**Conclusions:**

These findings demonstrate that GTS patients use a characteristic limbic suppression strategy. In contrast, control subjects use the voluntary sensorimotor circuits and the classical ‘stop’ network to suppress natural urges. The employment of different neural suppression networks provides support for cognitive behavioral therapy in GTS.

## Highlights

• Neural networks of tic suppression are specific and differ from blink suppression.
• Tourette patients employ a limbic suppression strategy to suppress tics.
• Controls use sensorimotor circuits and ‘stop’ networks for blink suppression.
• Objective task performance is highly recommended during functional MRI of tics.

## 1 Introduction

Gilles de la Tourette Syndrome (GTS) is a neuropsychiatric disorder defined by the presence of multiple motor and vocal tics. Tics typically develop during childhood and wax and wane over time (Singer, 2005). The first tics to develop in childhood usually encompass simple facial tics, for instance ocular tics or nose twitching (Jankovic, 1997). Ocular tics are present in almost all patients with GTS and include forceful eye blinking, eye rolling, or squinting (Karson et al., 1985; Martino et al., 2012). One of the key clinical features of tics is the ability to suppress the unwanted movement. Notably, ocular tics are the most difficult tics to suppress in GTS. Tics are often preceded by a premonitory sensation or an urge and tic execution may provide temporary relief (Singer, 1997). The urge to tic increases during tic suppression. Patients often report that the premonitory urge to tic increases during tic suppression, although tic self- rating by patients has been proven to be unreliable (Muller-Vahl et al., 2014). It is assumed that the relief from premonitory urges functions as a negative reinforcer, which leads to tic maintenance (negative reinforcement model) (Beetsma et al., 2014; Brandt et al., 2016; Capriotti et al., 2014). During adolescence the awareness of premonitory urges increases with increasing age, and there is some evidence that this improves the ability to suppress tics (Banaschewski et al., 2003). Although it is true that the awareness of urges increases with age, it is unclear whether this increases the ability for tic suppression. There is even some conflicting evidence that urges and tic inhibition are not directly related (Ganos et al., 2012).

The pathophysiology of GTS remains unclear (Ganos et al., 2013). GTS is hypothesized as a disorder of inhibition, in which patients have impaired capability to restrain their urges to tic. Based on post-mortem pathology and imaging studies a primary dysfunction of the basal ganglia (BG) and their output pathways via the corticostriatal circuits is suggested (Bohlhalter et al., 2006; Peterson et al., 1998; Wang et al., 2011; Worbe et al., 2012). The clinical observation that GTS patients are capable to temporarily overrule their tics by suppression, while their disorder can in essence be regarded as a disinhibition of motor control, is a poorly understood paradox.

Few neuroimaging studies have investigated the mechanisms by which patients are capable to temporarily suppress tics (Ganos et al., 2014a; Kawohl et al., 2009; Peterson et al., 1998). Peterson and colleagues studied 22 GTS patients during suppression of tics during functional Magnetic Resonance Imaging (fMRI) showing increased activity of the caudate nucleus, and a decrease of activity in the putamen, globus pallidus and thalamus (Peterson et al., 1998). Another study in a single GTS patient, found the anterior cingulate cortex (ACC) to be active during tic suppression (Kawohl et al., 2009). A third study found in 14 GTS patients increased activity of the left inferior frontal gyrus as a sole finding during tic suppression compared to the release of tics (Ganos et al., 2014a). These papers, however, lack comparison to control subjects. As a model to study tic suppression in healthy controls, several studies have investigated the suppression of natural urges such as normal eye blinking. Intuitively, the premonitory tension and urge experienced just prior to tic onset appear to be similar to the somatosensory tension experienced during sustained voluntary suppression of eye blinks (Mazzone et al., 2010). Lerner and colleagues found a central role for the insula and the ACC in blink suppression (Lerner et al., 2009). Mazzone and colleagues observed increased activation of the right middle frontal gyrus (Brodmann area, BA 9), left dorsal anterior cingulate cortex (BA32) and the bilateral superior frontal gyrus (BA10) during blink suppression in GTS compared to control subjects (Mazzone et al., 2010). However, it remains unclear to what extent this increased frontostriatal activity in GTS is specific for tic suppression.

The current study is the first to directly compare the neural correlates of suppression of ocular tics in patients with GTS with the suppression of eye blinks in healthy controls. Another novelty of this study is that we ensure a true comparison of motor output suppression versus release during task performance since we incorporate task performance, as objectively measured with the eye-tracker, in the analyses of the fMRI. This also enables us to compare the participants' self-report measures of suppression ability with their objective ability to follow task instruction.

Our first objective is to explore the neural correlates of tic suppression in GTS. We hypothesize that GTS patients during suppression will demonstrate increased activation in the caudate nucleus and ACC. Second, we aim to explore the neural correlates of blink suppression as a model of the suppression of natural urges in control subjects, and we hypothesize that healthy control subjects demonstrate increased activation of the insula and the ACC. Our third objective is to compare the suppression strategy of tics in GTS patients with blink suppression in healthy control subjects. We hypothesize that frontostriatal activity is increased in GTS compared to controls during suppression. To validate our task and confirm previous findings on tic generation we also investigate tic release (Bohlhalter et al., 2006; Hampson et al., 2009; Neuner et al., 2014; Stern et al., 2000; Wang et al., 2011). Three separate processes are hypothesized to be active during tic release. The first is the prime tic generator (mediated by BG (Ganos et al., 2013)), the second mediates release of tic control (predominantly controlled by supplementary motor area (SMA) (Bohlhalter et al., 2006; Hampson et al., 2009; Wang et al., 2011), and a third process is responsible for tic execution (encompassing the sensorimotor system, consisting of the cerebellum, somatosensory and (pre)motor cortex (Bohlhalter et al., 2006; Hampson et al., 2009; Wang et al., 2011)). Thus, during release of tics we hypothesize that GTS patients show increased activity in the BG and sensorimotor system, in particular the SMA.

## 2 Materials and methods

### 2.1 Participants

Sixteen patients fulfilling DSM-IV-TR criteria of GTS participated in this study. Twenty-two healthy controls without neurological or psychiatric conditions and without psycho-active medication were included. Patients were recruited from a previously performed video and EEG study, measuring the Bereitschaftspotential (BP) prior to the onset of motor tics (for a full description of the participants see (van der Salm et al., 2013a; van der Salm et al., 2012; van der Salm et al., 2016)). Inclusion criteria for patients the presence of both eye and motor tics and the ability to suppress and release their motor and ocular tics on demand. The ability to suppress tics was tested and clinically judged during the previous EEG and video studies. (van der Salm et al., 2013a; van der Salm et al., 2012; van der Salm et al., 2016) We excluded one patient because of technical eye-tracker malfunction. Data of 15 patients and 22 controls were analyzed on task performance (see below). Patients and controls were matched at group level on gender, age, education level (Verhage, 1964) and handedness (Oldfield, 1971) (see Table 1 for demographic characteristics). Prior to scanning medical history and psychiatric history or current psychiatric symptoms (exclusion criteria) were inquired in healthy control subjects. Psychiatric co-morbidity in patients was assessed with the MINI plus (van Vliet et al., 2000). Three out of fifteen GTS patients were diagnosed with co-morbid OCD, and one patient with co-morbid ADHD. Thirteen patients were medication free during testing. The GTS patient with ADHD was on methylphenidate which was continued during scanning. Two patients were on benzodiazepines (alprazolam and clonazepam) from which they abstained for >24 h before scanning. The local medical ethics committee approved the study. Written informed consent was obtained from all participants.

**Table 1:** Demographic and clinical characteristics of patients and controls.

Characteristics	GTS patients (*n* = 15)	Control subjects (*n* = 22)
Age in years (SD)	34.8(8.9)	42.7(15.1)
Gender (M/F)	13/2	13/9
Education (SD)	5.3 (0.8)	5.4 (1.2)
Comorbidity		–
OCD	3	–
ADHD	1	–
Psycho-active medication (%) during scanning	1 (7%)	0 (0%)

Legend: ADHD = attention deficit hyperactivity disorder; F = female; GTS = Gilles de la Tourette syndrome M = male; OCD = obsessive compulsive disorder.

There was no significant difference between groups in age (*p* = .262 Mann Whitney *U* test), gender (*p* = .075 chi-squared test) or educational level (*p* = .453; Mann Whitney U test). Education was scored in the Dutch classification system according to Verhage, encompassing 7 categories. 1 = did not finish primary school, 2 = finished primary school, 3 = did not finish secondary school, 4 = finished secondary school, low level, 5 = finished secondary school, medium level, 6 = finished secondary school, highest level, and/or college degree, 7 = university degree. (Verhage, 1964).

### 2.2 Behavioral task

The task in the scanner consisted of two alternating blocks. During ‘suppress’ blocks, controls were instructed to suppress blinking and patients to suppress ocular tics but not blinks. During ‘release’ blocks, controls were allowed to blink and patients to exhibit their tics. Prior to scanning, participants practiced the tic or blink suppression task outside the scanner.

### 2.3 Subjective ratings

Suppression and release of blinking and tics was monitored subjectively with subjects' feedback of their performance on a ten point rating scale (0 indicating complete inability of suppression and 10 excellent suppression ability) during debriefing directly after the completion of the task. In order to assess the accuracy of subjective measurements of the ability of suppression, we compared subjective measurements of the 10-point rating scale, with the objective measurements (number of tics per suppression/release) using the eye-tracker recordings.

### 2.4 Blink/tic detection

Specifications of the eyetracker system are found in the supplement. Blink or tic onset times were determined by measurement of the pupil diameter in every video frame (1 frame = 40 ms). Upon absence of the pupil, the pupil diameter was defined as ‘undetected’. With a threshold of 3 undefined values, a custom written MATLAB script (version 7.8, The Mathworks, Natick MA) reported the onset time and duration of all ‘undetected pupil’ moments. Subsequently, to discern tics from blinks, video recordings of ‘undetected pupil’ moments were clinically judged and scored by two separate raters (SvdS, EB). Based on a previous EEG and video study of the same GTS patient sample we characterized for each patient the phenomenology of individual tics or blinks prior to scoring of eyetracker video recordings (van der Salm et al., 2013a; van der Salm et al., 2012; van der Salm et al., 2016). Events detected by the eye-tracker were subdivided into the categories based on phenomenology as: blinks, ocular tics or false positive detections (other ocular movements e.g. staring with gaze deviation or sleepiness). In case of rater disagreement, events were discussed and assigned after between-rater consent. In addition to the eye blink recordings, the entire patient was monitored during scanning by means of closed-circuit television (CCTV) video registration depicting the trunk, arms and legs during scanning. This was used as means to clinically observe patients and the amount of body tics they had. It was only visually inspected during offline analysis and used during the interpretation of the movement parameters and to observe bodily tics in relation to the movement parameters during image analysis. Video recordings of the patient in the scanner and the eye-tracker recording were all synchronized in time with the MRI scans.

### 2.5 Behavioral analyses

Baseline characteristics were compared with non-parametric Mann Whitney *U* tests and in the case of dichotomous variables chi-squared tests. Task performance comparisons between subjective and eye-tracker ratings were investigated using Pearson correlation coefficient (Pearson's r). A significance threshold of *p* < .05 was applied using the IBM Statistical Package for the Social Sciences (SPSS) software.

### 2.6 Image analyses: performance - adapted fMRI analysis

Images were acquired on a 3 Tesla Philips Intera scanner (Intera, Philips Healthcare, Best, the Netherlands) and details are listed in the supplement. Imaging data were analyzed with the Statistical Parametric Mapping software (SPM5: www.fil.ion.ucl.ac.uk/spm; London) for Matlab. Functional images were slice-time corrected, spatially realigned, normalized into the standard space of the MNI-152 Template brain, and smoothed with an 8-mm Gaussian kernel (Friston et al., 1995).

**Fig. 1:**
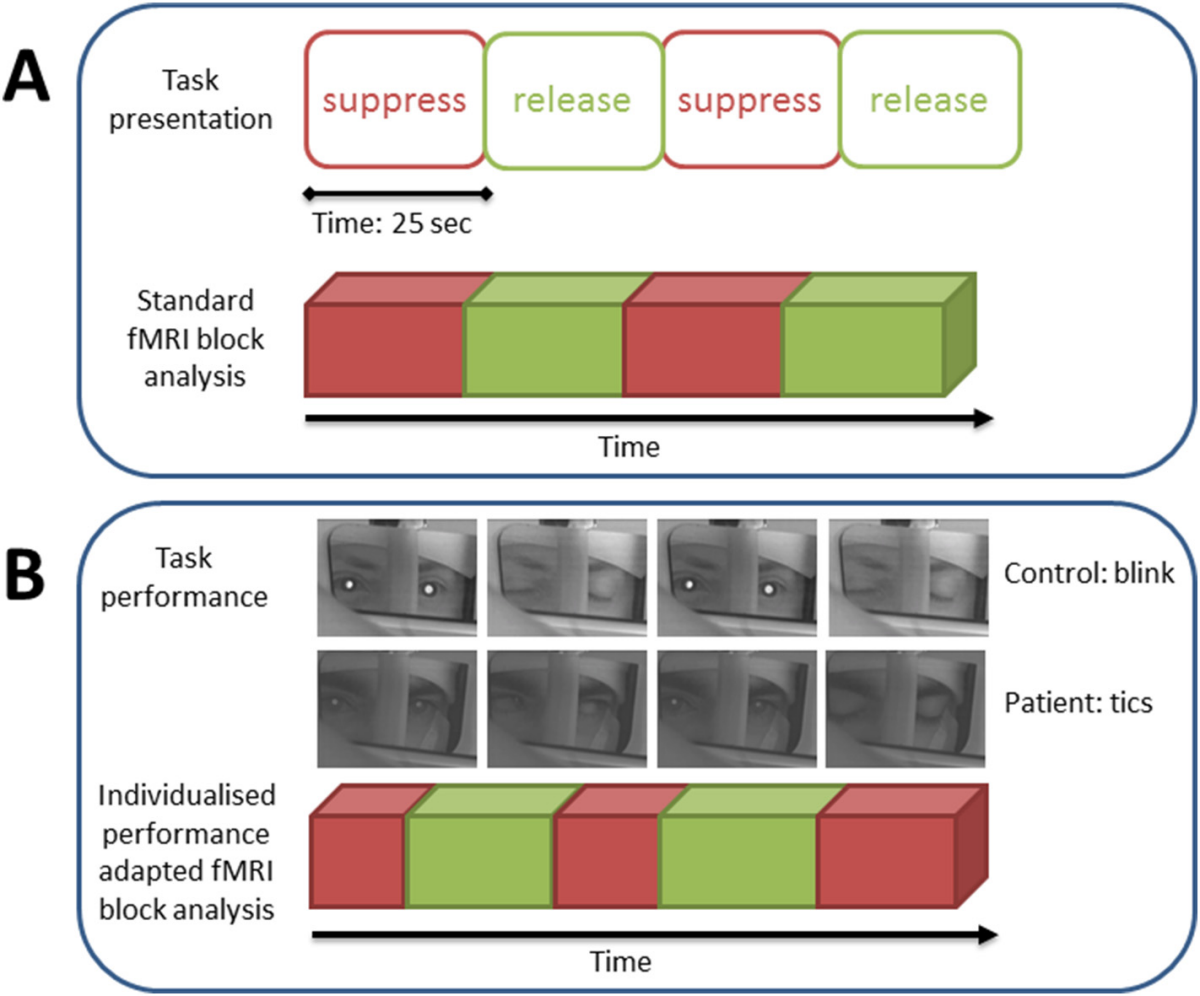
Experimental design of this study. Fig. 1 displays the difference of standard fMRI analysis of the block design (Fig. 1A) and the performance- adapted fMRI analysis as used in this study (Fig. 1B). In the standard block design one assumes that subjects perform the task exactly as instructed by the research team. In the current study, patients are instructed to suppress tics and the controls to suppress blinking. During the release condition patients and controls may release tics and blinks. In contrast, in the current study we used performance-adapted block design (Fig. 1B), that incorporates the task execution, as measured by the eye-tracker, into the fMRI analysis. In all participants, duration of the blocks was adapted based on the performance of the task as measured with the eye-tracker (performance- adapted fMRI analysis). The suppression block ended at the first blink (controls) or tic detected based on the eye-tracker recording. In the supplemental videos (available online) the task execution of a control subject and Tourette patient can be found and the videos demonstrate the clinical difference of blinks and tics.

In the single-subject analyses, the General Linear Model (GLM) consisted of a block regressor encoding the ‘suppress’ vs. ‘release’ condition. Alternating blocks lasted for 25 s (10 scans) and were repeated 11 times. For fMRI analysis, the duration of the blocks was adapted based on the performance of the task as measured with the eye-tracker (performance-adapted fMRI analysis). The suppression block ended at the first blink (controls) or tics (GTS) detected by the eye-tracker recording. Fig. 1 displays the difference of performance-adapted fMRI analysis with standard fMRI analysis of the block design (Fig. 1A), in which one assumes the subjects perform the task exactly as presented. In contrast, performance-adapted fMRI analysis (Fig. 1B) incorporates the task execution into the fMRI analysis. The mean duration of performance-adapted blocks was 29,97 in GTS (SD 5,34) and 27,75 s in controls (SD 2,71).

All performance- adapted block regressors were convolved with the canonical Hemodynamic Response Function to model the Blood‑oxygen-level dependence (BOLD) response. To correct for head motion-related artifacts, rotation and translation parameters obtained from the spatial realignment were added as covariates. In addition, we used scan-nulling regressors into the GLM analysis to model changes in the BOLD-signal associated with large inter-scan motion events (head movement) (Lemieux et al., 2007; van der Salm et al., 2013b). In case subjects' movement was over 0.2 mm/scan, that scan and three subsequent scans were rejected. If an overall threshold of 25% rejected scans was exceeded the patient was excluded from further image analyses. This threshold was defined prior to scanning of the subjects and the image analyses.

In the second-level analyses, the first-level contrast images for suppress vs. release were entered into a random effects analysis in order to detect voxel-wise differences in BOLD response between patients and controls. To control for multiple comparisons, statistic images were assessed for cluster-wise significance using a cluster-defining threshold of *p* = .005, and *p* < .05 corrected for multiple comparisons (family-wise error, FWE); the critical cluster size will be reported per contrast.

To investigate the disorder specific neural correlates of suppression vs. release of GTS and the neural correlates of the suppression vs. release of eye blinks in controls, we first perform a within- group analysis of suppression per group. To investigate the differences in neural networks involved in suppression of tics and blinks, we performed a between group analysis (GTS > controls, controls > GTS).

## 3 Results

### 3.1 Behavioral results

Illustrative videos of the eye-tracker recording of a patient alternating suppression and release of tics and a control subject suppressing and releasing blinks are provided in the supplement. All included patients had ocular tics, mostly with tonic contractions of eye musculature, eye deviations or eye rolling, and not so much blinking tics, which would have been difficult to differentiate from normal blinking.

Analysis of tasks behavior showed the following. During suppression patients had a significant reduction in ocular tics as compared to the release condition (*p* = .003), signifying that they performed as instructed. In controls, blinking rate significantly decreased during the suppression condition, relative to the release condition (*p* < .001). Although patients were instructed to suppress tics, patients also showed a significant reduction in blinks during suppression compared to the release condition (*p* = .006). See the supplement for details of blink and tic frequencies.

Next, we compared the subjective (self-rating) and objective (eye-tracker) ability of suppression during the task within subjects. Four controls and two patients were unable to score their suppression performance on a 10-point scale. Self-ratings of blinking and tic frequencies differed from their actual performances as measured with eye tracking across patients and controls with no significant correlations between subjective ratings and actual task performance (patients r^2^ = −0.375, *p* = .207; controls r^2^ = 0.012, *p* = .963). Patients had a mean self-rating of 7,98 (SD 1,04) and the control subjects of 7,3 (SD 1,5) for the ability of suppression during the entire task.

### 3.2 Functional imaging results

Four patients and two controls were excluded from further analysis due to excessive head motion. Thus, subsequent imaging analysis could be conducted in eleven GTS patients and 19 control subjects (mean age of 33.8 and 41.3 years respectively). Exclusion of the single patient on methylphenidate did not change group findings, and the patient was therefore retained in the analysis.

#### 3.2.1 Within-group effects

**Fig. 2:**
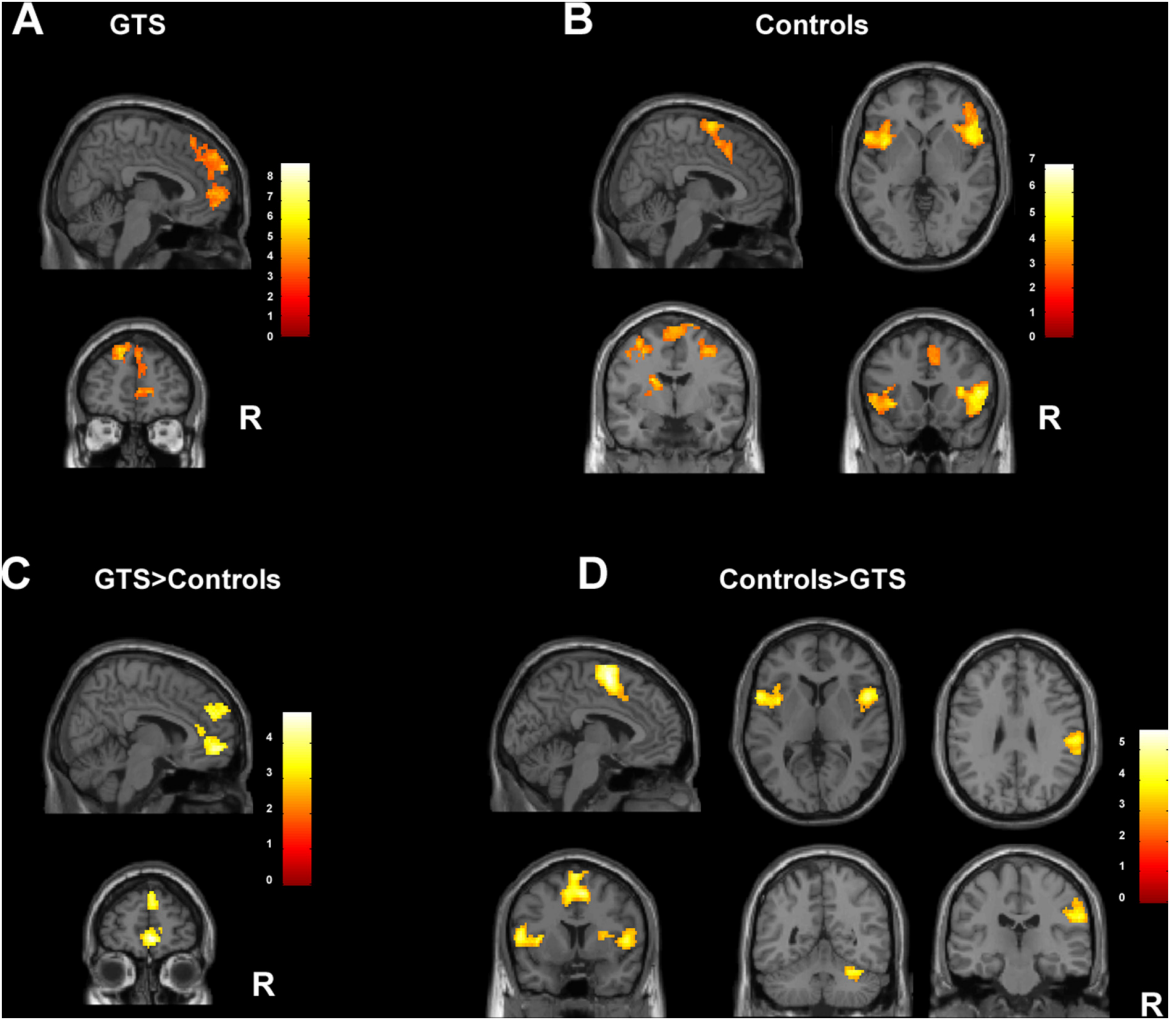
Overview of the main findings of the suppression task. 2A Group specific neural correlates of suppression in the GTS patients. Suppression of tics by GTS patients (within group analysis) led to increased activity compared to release in the bilateral frontal eye fields, right anterior prefrontal cortex, right dorsolateral prefrontal cortex. Not all activations are shown in the fig. 2B Group specific neural correlates of suppression of the control subjects. Suppression of eye blinks of the control subjects (within group analysis) resulted in increased activity in the bilateral inferior frontal gyrus/insula, premotor cortex and SMA, right ventrolateral prefrontal cortex, the left putamen and caudate and bilateral inferior parietal cortex right anterior prefrontal cortex. 2C Differences between groups during suppression. Comparison of increased activity in GTS patients compared to control subjects during suppression (between group comparison), depicting the increased activity in the right anterior prefrontal cortex, ACC, the left frontal eye fields, right superior frontal cortex and bilateral dorsolateral prefrontal cortex. 2D Differences between groups during suppression. Comparison of increased activity in control subjects compared to GTS patients during suppression (between group comparison), depicting the activity of the bilateral SMA and CMA, bilateral insula and ventrolateral prefrontal cortex, right putamen, and cerebellum. R = right hemisphere. BA = Brodmann area. Sagittal, coronal and axial planes are shown. *P* values all corrected for multiple comparisons.

Analysis of tic suppression versus release within the GTS patients demonstrated increased activity in bilateral middle temporal cortex (BA21), bilateral frontal eye fields (BA8), left inferior occipital gyrus (BA18), right anterior prefrontal cortex (BA10), right dorsolateral prefrontal cortex (DLPFC, BA46) and right inferior parietal cortex (BA40). Analyses of blink suppression within healthy controls showed increased activity of the bilateral pars opercularis of the inferior frontal gyrus (BA44) extending to insula (BA13), bilateral premotor cortex and SMA (lateral and medial BA6), right inferior frontal gyrus (BA47), left putamen and caudate, right somatosensory cortex (BA2) and bilateral inferior parietal cortex (BA40), and right anterior prefrontal cortex (BA10). See Table 2 and Fig. 2A-B.

**Table 2:** Group specific neural correlates of suppression.

Group	Side	Region of activation	BA	K e	MNI Coordinates			Z score	P value
					X	Y	Z		
GTS	L	Middle temporal cortex	21	216	−60	−20	−8	4.58	<0.001
	B	Superior frontal gyrus, frontal eye fields	8	702	−11	47	46	4.02	<0.001
	L	Inferior occipital gyrus	18	152	−23	−90	−14	3.88	0.005
	R	Middle temporal cortex	21	169	58	−4	−20	3.85	0.002
	R	Lateral temporal cortex	21	232	65	−17	−2	3.77	<0.001
	R	Anterior prefrontal cortex	10	151	32	57	7	3.74	0.005
	R	DLPFC	46	117	56	33	16	3.56	0.023
	R	Anterior prefrontal cortex, dorsal anterior prefrontal cortex	10,32	283	6	55	−8	3.45	<0.001
	R	Inferior parietal cortex	40	140	47	−46	37	3.16	0.008
C	L	Pars opercularis of the inferior frontal gyrus, Insula	44	958	−54	11	7	5.05	<0.001
			13		−38	9	1		
	R	Premotor cortex	6	343	28	−2	61	4.95	0.001
	R	Insula	13	975	34	22	10	4.73	<0.001
		Inferior frontal gyrus (VLPFC)	44, 45		54	15	10		
					47	20	1		
	L	Putamen	–	188	−25	−6	7	4.54	0.022
		Caudate			−13	−2	14		
	R	Somatosensory cortex	2	524	65	−24	31	4.21	<0.001
		Inferior parietal cortex	40		43	−35	46		
	L	Parietal operculum	40	222	−38	−44	55	4.03	0.009
	B	SMA	6	688	8	0	70	4.00	<0.001
					−8	0	61		
	L	Premotor cortex	6	246	−32	−6	55	3.89	0.005
	R	Anterior prefrontal cortex	10	225	30	51	37	3.43	0.008

Table 2 lists the areas of statistically significant activations during the suppression condition per group, indicating the group specific neural correlates as tested with within-group analysis. Results are shown of 11 patients suppressing tics but not blinks and 19 healthy control subjects suppressing blinks.

BA = Brodmann area; B = bilateral; C = Controls; DLPFC = dorsolateral prefrontal cortex; GTS = Gilles de la Tourette syndrome patients; Ke = cluster extent; L = left; R = right; SMA = supplementary motor area; VLPFC = ventrolateral prefrontal cortex.

Cluster defining threshold = 0.005, *p* value < .05 (corrected for multiple comparisons. FWE = family-wise error).

**Fig. 3:**
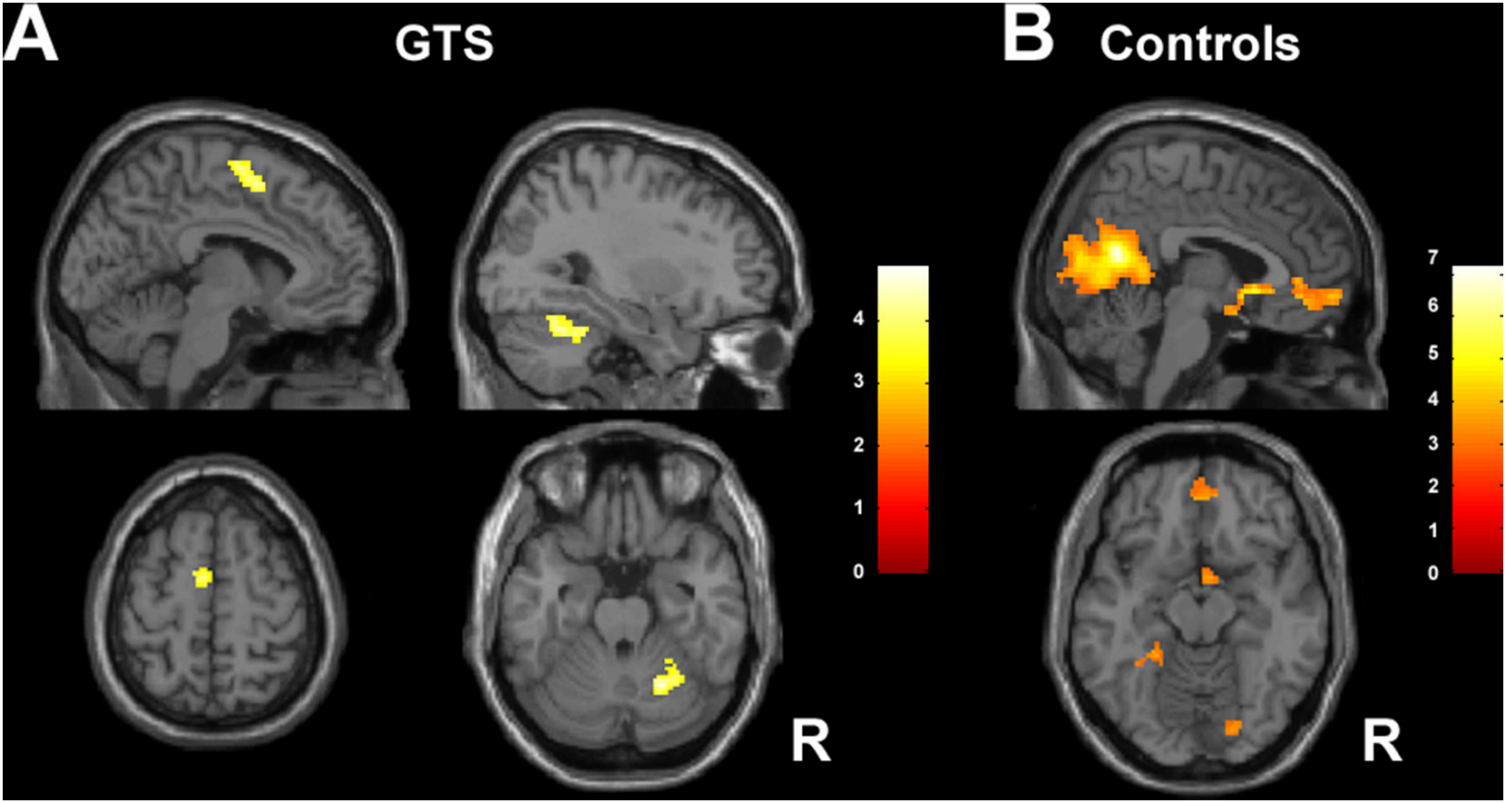
Overview of the main finding of the release condition. Group specific findings during release of tics in patients and release of blinks in controls are shown. 3A depicts the release of tics in GTS patients (within group analysis). It shows the left SMA and right cerebellum. 3B demonstrates the release of blinks in controls (within group analysis). It shows increased activity of the bilateral parahippocampal gyrus, posterior cingulate cortex and precuneus, the right subcallosal cingulate gyrus and the ventral anterior cingulate and the anterior prefrontal cortex and superior frontal cortex). R = right hemisphere. Sagittal and axial planes are shown.

Analysis of tic release versus suppression in GTS patients demonstrated increased activity in right cerebellum and left SMA (BA6). Analysis of blink release versus suppression within controls demonstrated increased activity of the bilateral parahippocampal gyrus, posterior cingulate cortex and precuneus (B30, 23, 30), right subcallosal and ventral anterior cingulate gyrus (BA25, 32), anterior prefrontal and superior frontal cortex (BA10). See Table 4 and Fig. 3A-B.

#### 3.2.2 Between-group effects

During suppression, GTS patients showed increased activation of the right anterior prefrontal cortex (BA10) and ACC (BA32), left frontal eye fields (BA8) and premotor cortex (BA 6), and right superior frontal and dorsolateral prefrontal cortex (BA10 and BA 9) compared with controls.

Control subjects showed more activation of the bilateral SMA and cingulate motor area (CMA; BA6, 32), left insula (BA13), right putamen and right pars opercularis of the inferior frontal gyrus (BA44), right cerebellum (*p* = .044) and right inferior parietal cortex (BA40) in comparison to GTS patients. See Table 3 and Fig. 2C-D.

**Table 3:** Differences between groups during suppression.

Group	Side	Region of activation	BA	K e	MNI Coordinates			Z score	P value
					X	Y	Z		
GTS > C	R	Anterior prefrontal cortex	10	470	6	53	−5	4.12	<0.001
		ACC	32		10	46	−2		
	L	Superior frontal cortex, frontal eye fields	8	266	−16	51	43	4.06	0.007
		Premotor cortex	6		−23	22	58		
	R	Anterior prefrontal cortex	10	205	6	62	31	3.69	0.028
		DLPFC	9		6	46	31		
C > GTS	B	SMA	6	1194	−5	0	67	4.60	<0.001
		CMA	32		8	7	52		
	L	Insula and frontal operculum	13	460	−36	7	7	3.71	<0.001
	R	VLPFC and insula	44, 45, 13	418	58	11	10	4.18	<0.001
		Putamen	–		28	4	13		
	R	Cerebellum	–	186	32	−55	−26	3.78	0.044
	R	Inferior parietal cortex	40	296	60	−24	28	3.76	0.004

Table 3 lists the areas of statistically significant activations that differ between GTS patients and controls during suppression, as tested with between-group analysis. Results are shown of 11 patients suppressing tics but not blinks and 19 healthy control subjects suppressing blinks.

ACC = anterior cingulate cortex, BA = Brodmann area; B = bilateral; C = controls; CMA = cingulate motor area; DLPFC = dorsolateral prefrontal cortex; GTS = Gilles de la Tourette syndrome patients; Ke = cluster extent; L = left; R = right; SMA = supplementary motor area; VLPFC = ventrolateral prefrontal cortex.

Cluster defining threshold = 0.005, p value <.05 (corrected for multiple comparisons. FWE = family-wise error).

**Table 4:** Group specific findings during release of tics in patients and release of blinks in controls.

Group	Side	Region of activation	BA	K e	MNI Coordinates			Z score	P value
GTS	R	Cerebellum	–	257	23	−59	−23	3.41	<0.001
	L	SMA	6	126	−5	−4	64	3.35	0.015
C	B	Parahippocampal gyrus	30	4248	−16	−46	−2	4.93	<0.001
		Precuneus	23		8	−61	19		
		Posterior cingulate cortex	30		14	−55	7		
	R	Subcallosal cingulate gyrus	25	168	3	13	−5	4.07	0.039
	R	Ventral anterior cingulate	32		3	22	−8		
	B	Anterior prefrontal cortex	10	271	8	40	−8	3.46	0.003
					−5	64	−8		

Table 4 lists the areas of statistically significant activations during the release condition per group, indicating the group specific neural correlates of release as tested with within-group analysis. Results are shown of 11 patients releasing tics and 19 healthy control subjects releasing blinks.

BA = Brodmann area; B = bilateral; C = Controls; GTS = Gilles de la Tourette syndrome patients; Ke = cluster extent; L = left; R = right; SMA = supplementary motor area.

Cluster defining threshold = 0.005, *p* value < .05 (corrected for multiple comparisons. FWE = family-wise error).

## 4 Discussion

Our study is the first to directly compare the neural circuits involved in ocular tic suppression and release in GTS patients and blink suppression and release in healthy controls. During suppression, our main findings are prominent activation of the dorsal ACC in patients contrary to the bilateral SMA and CMA activations in healthy control subjects. These findings imply different suppression strategies within different neural circuits. In particular, the limbic circuit is employed in GTS during tic suppression, while the sensorimotor circuit plays a key role in suppression of blinks in control subjects. Moreover, control subjects employ the classical ‘stop’ network, located in the right ventrolateral prefrontal cortex (VLPFC, and BA 44 and 45).

### 4.1 Performance and behavioral results

The combination of an eye-tracker and fMRI is an innovative multi-modal approach enabling the incorporation of the actual task performance in the fMRI design. Behaviorally, both controls and patients successfully suppressed blinks or tics. Controls significantly decreased blinking rate during suppression compared to spontaneous blinking. An interesting finding is that participants' self-rating of their suppression ability did not correlate to their objective performance as measured with the eye-tracker. This is in line with the commonly held misbelieve of a tic rebound and suggest that in general self-rating and objective measurement differ in GTS patients (Muller-Vahl et al., 2014).

We recommend future studies to employ objective monitoring of task performance instead of solely relying on subjective measures during fMRI.

### 4.2 Neural correlates of tic versus blink suppression

During suppression, the main findings of our study are prominent activation of the dorsal ACC in GTS patients in contrast to right VLPFC, bilateral SMA and CMA activations in healthy control subjects. These main findings imply different underlying neural networks in tic and blink suppression related to altered activation of the different loops of the corticostriatal circuit (CSC). The CSC is commonly divided into the sensorimotor, associative and limbic circuits, which are involved in the control and selection of goal-directed motor, cognitive and motivational behavior respectively (Alexander et al., 1986; Delong and Wichmann, 2007; Mink, 2003; Obeso et al., 2014).

Firstly, our results demonstrate the involvement of the sensorimotor circuit in the suppression of eye blinks in healthy controls, whereas this region was not activated in GTS patients during tic suppression. We argue here that this increased activation reflects the somatosensory tension or urge to blink that builds up in control subjects during suppression. In GTS patients an urge is also present during suppression, but we hypothesize that GTS patients are more used to the presence of an urge during longer periods of suppression resulting in less somatosensory cortex activation. Further, in healthy controls compared to GTS we found increased activation in the SMA and CMA during suppression. The CMA is responsible for facial muscle coordination. We hypothesize that healthy controls used co-contraction of the eye musculature to prevent blinking. By means of clinical observation (CCTV and eyetracker), we observed squinting in control subjects, operationalized as slight tonic contraction (squinting) of the eye musculature during the suppression task. Thus, it appears that the healthy controls utilize a motor suppression paradigm in contrast to GTS patients. Further, it is possible that increased SMA activation in GTS patients during tic release is driving the group results in this region. Possibly, reflecting involvement of the SMA in selecting to execute tics.

Secondly, we found that the DLPFC, a region part of the associative loop is involved in tic suppression. The DLPFC is thought to regulate self-control by inhibiting the premotor cortex to forestall planned motor actions (Ramnani and Owen, 2004) (Devinsky et al., 1995).

Thirdly, our results confirm the involvement of prefrontal structures of the limbic loop in the suppression of tics. Recently it was suggested in a resting state fMRI study that the orbitofrontal cortex is primary involved in tic suppression in GTS patients (Ganos et al., 2014a). The orbitofrontal cortex has been implicated in processes that involve the motivational or emotional value of incoming information and the integration of this information to guide response selection, suppression and decision making (Ramnani and Owen, 2004). Our results indicate overlapping involvement of the orbitofrontal cortex in both the suppression of natural urges as well as tics. We found that rather than the OFC the dorsal ACC is specifically involved in the volitional suppression of tics in GTS patients. This is in line with studies suggesting that the ACC controls the decision *not* to move (Devinsky et al., 1995). Cognitive behavioral therapeutic strategies, such as habit reversal training (HRT), have proven effective in GTS (Piacentini et al., 2010; Verdellen et al., 2007). In HRT, GTS patients are taught to recognize the urge preceding the tic and taught to try to alleviate the urge by an alternative action rather than a tic. Future studies may aim to see if altering ACC activation for instance in a neurofeedback paradigm clinically improves tic suppression and HRT success.

Finally, blink suppression in control subjects was associated with recruitment of regions of the ‘classical’ stop network, i.e. bilateral VLPFC extending tot anterior insula. The VLPFC is a critical region for inhibiting a (preplanned) motor response (Aron et al., 2007). VLPFC activation was significantly increased in controls versus patients during suppression. This is a novel finding since previous studies did not find abnormalities in the right VLPFC during motor inhibition in GTS patients compared with controls (Ganos et al., 2014a; Ganos et al., 2014b; Thomalla et al., 2014). The observed anterior insula activation may indeed also be related to motor inhibition. Alternatively, activation of the insula as well as the putamen in controls may imply an increase of interoception or self-awareness, possibly due with unpleasant feelings associated with blink suppression (Craig, 2009). We also found some evidence for overlap between the neural circuits involved in the suppression of natural urges (blink) and tics. Behaviorally, patients also significantly blinked less when they suppressed tics contrary to the task instruction. A possible explanation is that increased attention of the participants to correct task execution decreased blinking rate, which has been extensively demonstrated in healthy controls (Nakano et al., 2013). An alternative explanation may be that activation of the tic suppression brain regions resulted in blink suppression in GTS patients.

To summarize our findings, the tic suppression networks appear to be mainly limbic and associative. This is in line a recent study investigating the effect of thalamic deep brain stimulation in GTS (Jo et al., 2018). Ultimately, GTS might be best considered as a social decision-making network disorder, instead of a BG disorder, as was recently proposed because patients can choose to alter or inhibit tics in social contexts(Albin, 2018). Our study and this novel interpretation as a disorder of social decision-making call for new investigations of the these networks in GTS patients.

Our findings on the networks involved in tic suppression and release may also have therapeutic implications. For GTS patients with severe and medically refractory tics, deep brain stimulation (DBS) is considered a therapeutic option and proven effective (Martinez-Ramirez et al., 2018). Based on the phenotype of the most problematic tic a target for stimulation is chosen, which could be the thalamus, globus pallidus, anterior limb of the internal capsule or nucleus accumbens. Issues that remain unresolved include selection of appropriate brain target for individual symptoms. A recent study of intraoperative functional MRI demonstrated that thalamic stimulation in GTS patients has widespread effects on the frontostriatal, limbic, and motor networks. Motor tic reduction was correlated with suppression of motor and insula networks due to thalamic stimulation, while suppression of frontal and parietal networks correlated with vocal tic reduction (Jo et al., 2018). In contrast, our study primarily found a limbic suppression strategy in GTS patients suppressing ocular tics. Therefore, future studies on the optimal DBS target for specific tics are needed, and those studies need to take network effects into account.

### 4.3 Neural basis of voluntarily tic release

Analysis of neural activation during tic release further replicates previous findings suggesting that the SMA is involved in the voluntary release of tics in GTS (Hampson et al., 2009; Mazzone et al., 2010). Our fMRI results converge with the presence of the Bereitschaftspotential (BP) which precedes voluntary actions, which we showed previously to precede the motor tics by about 1000 ms in the same GTS patients (van der Salm et al., 2012). Based on our findings and the literature we postulate that the release of tics in our sample is indeed a voluntary action (Cunnington et al., 2003). The SMA was previously therapeutically targeted by means of repetitive TMS. It was demonstrated by several repetitive TMS studies that stimulation of the SMA led to a decrease in tic severity (Chae et al., 2004; Kwon et al., 2011; Mantovani et al., 2007; Mantovani et al., 2006).

The second key structure in the release of tics as found in this study is the cerebellum. The right cerebellum is hyperactive during suppression, again indicative of the involvement of the voluntary motor control circuit in the suppression of eye blinks. The particular role of the cerebellum in tics is relatively underreported in literature. The cerebellum was found in previous studies on tic generation and was likely involved in tic execution rather than tic generation (Bohlhalter et al., 2006; Lerner et al., 2009; Wang et al., 2011). An interesting study studying the effects of DBS in both globus pallidus internus (GPi) GTS patients found that effective stimulation resulted in flow reductions in the cerebellum and increases in the central and frontal cortex, specifically encompassing the SMA (Haense et al., 2016).

The cerebellum was found in all previous studies on tic generation and metabolic brain networks. (Bohlhalter et al., 2006; Lerner et al., 2009; Wang et al., 2011) (Haense et al., 2016; Pourfar et al., 2011).

It is difficult to distinguish if the activity in the cerebellum is due to the tic execution, thus secondary involved, or causal in the neural network of tic generation; this needs to be addressed by future studies.

In the current study, contrary to previous studies on tic generation, we did not find increased activation of the BG during the release of tics in patients (Bohlhalter et al., 2006; Peterson et al., 1998; Wang et al., 2011; Worbe et al., 2012). The results of the current study, however, rather imply that the BG are not the prime player or the epicenter of the release of tics, but rather follow cortical command, which was suggested previously in an extensive review (Ganos et al., 2013).

Another point to consider in the interpretation of our findings during suppression is the different generators for tics (striatum, as shown in animal models) and blinks (brainstem) (Hashemiyoon et al., 2017). Because these structures and their connected networks are employed to generate tics, this may in part explain the difference in suppression networks between healthy controls and GTS patients.

To end, in control subjects we found an increased activity of the bilateral occipital lobe (BA 18,23), precuneus, and posterior cingulate cortex during the release of blinks, which is concordant with blink (patho)physiology (Nakano et al., 2013).

### 4.4 Strengths and limitations

Strength of our study is the inclusion of a homogeneous group of GTS patients with predominant ocular tics and limb motor tics thorough phenotyping based on detailed clinical examination and BP testing (van der Salm et al., 2013a; van der Salm et al., 2012; van der Salm et al., 2016). We are aware, however, that our strict inclusion of patients with primarily motor tics and not vocal tics (suppression of which cannot be monitored during scanning) may impact the generalizability of our results to the full GTS clinical spectrum (which includes echolalia, coprolalia and patients without the ability of suppression). As noted above, another major strength of our study constitutes our explicit focus on the performance monitoring. Our data seem to suggest that methods employed by previous studies, such as self-reports and online analysis instead of offline video analysis (Neuner et al., 2007; Yoon et al., 2005) impair adequate measurement of brain activation related to tic suppression and release.

In theory, premonitory urges build up during the suppression of tics, and it could be argued that part of our findings during suppression might be caused by urges. On the contrary, however, in practice several studies found out that the link between urges and suppression is not so clear or even a false misbelief of patients (Ganos et al., 2012; Muller-Vahl et al., 2014). Rothwell and Edwards have alternately hypothesized that it would be simpler to consider tics as striatal habits that are to some extent modifiable by a volitional control (Rothwell and Edwards, 2011). Because stimulus–response associations exist at all levels of the sensorimotor system from spinal reflexes to striatal habits, they propose an alternative hypothesis that an urge to act is an expression of the interaction between these systems (stimulus & response, suppression & release), and that urge not a separate system apart from suppression (Rothwell and Edwards, 2011).

A limitation of our study is that the explicit request not to blink or tic during the fMRI suppression task and awareness of the monitoring by the research team might have increased the awareness of natural urges. Especially the urge to move during release of blinks in the healthy control subjects is an interesting finding. This resulted unfortunately in the exclusion of two healthy controls due to excessive motion. Another limitation of our study is the excessive (head) movement of participants in the scanner, and although motion correction was optimized using scan-nulling regressors, excessive motion resulted in exclusion of participants. Both patients as well as controls were excluded, decreasing both the number of patients in our study (*n* = 11) and the statistical power of our findings. It is a drawback of our study that some participants were unable to score their suppression ability verbally on a 10-point scale. However, the eye-tracker analysis enabled us to monitor performance during scanning.

Another limitation of our study is that active tic suppression in this study involved suppression of both tics of the eyes and motor tics, and therefore our findings during suppression and release cannot be further differentiated.

Although we did our best to clinically distinguish tics and blinks, with extensive clinical phenotyping of ocular and motor tics on video and EEG for each individual patient prior to scanning, it is a limitation of our study that the distinction between tics and blinks is not absolute. We acknowledge that it may very well be that the suppression vs release contrast in GTS patients is not exclusively related to tics, but also to blinks. Given the statistical strength of our findings however, it appears unlikely that in case that a small amount of tics may have been erroneously classified as blinks, or vice versa, would significantly change the findings of our study. This is because both tics and blinks occurred primarily in the release conditions, so the performance based data analysis block design would no change. Moreover, we feel there is no other methodological set up that will ensure 100% discrimination, especially because patients themselves have difficultly to distinguish tics and reliably rate tics (Muller-Vahl et al., 2014).

## 5 Conclusions

The current study demonstrates that the tic suppression network in GTS patients essentially differs from the inhibition network of natural urges in controls. Especially the limbic circuit is applied in GTS during tic suppression, while control subjects employ the classical ‘stop’ network to suppress eye blinks. Finally, the voluntary release of tics is primarily controlled by the SMA, corresponding to the Bereitschaftspotential that can be measured prior to the release of tics.

The following are the supplementary data related to this article.

Supplementary Video S1 A GTS patient releasing tics. Supplementary Video S1

Supplementary Video S2 A healthy control subject suppressing blinks (first 10 seconds) and releasing blinks (after 10 seconds). Supplementary Video S2
